# A 70% Ethanol Extract of Mistletoe Rich in Betulin, Betulinic Acid, and Oleanolic Acid Potentiated *β*-Cell Function and Mass and Enhanced Hepatic Insulin Sensitivity

**DOI:** 10.1155/2016/7836823

**Published:** 2016-01-14

**Authors:** Byoung-Seob Ko, Suna Kang, Bo Reum Moon, Jin Ah Ryuk, Sunmin Park

**Affiliations:** ^1^Korea Institute of Oriental Medicine, Daejeon 34054, Republic of Korea; ^2^Food & Nutrition, Obesity/Diabetes Center, Hoseo University, Asan 31499, Republic of Korea

## Abstract

We investigated that the long-term consumption of the water (KME-W) and 70% ethanol (KME-E) mistletoe extracts had antidiabetic activities in partial pancreatectomized (Px) rats. Px rats were provided with a high-fat diet containing 0.6% KME-E, 0.6% KME-W, and 0.6% dextrin (control) for 8 weeks. As normal-control, Sham-operated rats were provided with 0.6% dextrin. In cell-based studies, the effects of its main terpenoids (betulin, betulinic acid, and oleanolic acid) on glucose metabolism were measured. Both KME-W and KME-E decreased epididymal fat mass by increasing fat oxidation in diabetic rats. KME-E but not KME-W exhibited greater potentiation of first-phase insulin secretion than the Px-control in a hyperglycemic clamp. KME-E also made *β*-cell mass greater than the control by increasing *β*-cell proliferation and decreasing its apoptosis. In a euglycemic-hyperinsulinemic clamp, whole-body glucose infusion rate and hepatic glucose output increased with potentiating hepatic insulin signaling in the following order: Px-control, KME-W, KME-E, and normal-control. Betulin potentiated insulin-stimulated glucose uptake via increased PPAR-*γ* activity and insulin signaling in 3T3-L1 adipocytes, whereas oleanolic acid enhanced glucose-stimulated insulin secretion and cell proliferation in insulinoma cells. In conclusion, KME-E prevented the deterioration of glucose metabolism in diabetic rats more effectively than KME-W and KME-E can be a better therapeutic agent for type 2 diabetes than KME-W.

## 1. Introduction

There has been a marked increase in the prevalence of type 2 diabetes among individuals older than 40 years of age, and approximately 20% of people in Korea older than 65 years of age have type 2 diabetes, which is over two-fold times greater than the average rate for Organization for Economic Cooperation and Development countries [1]. Type 2 diabetes has been associated with incremental increases in insulin resistance due to Westernized lifestyles and diets [1–3]. Among Caucasians in Western countries, a high-fat diet leads to hyperinsulinemia, which is intended to compensate for increased insulin resistance [3]; however, it has been reported that Asians do not have a sufficient capacity for insulin secretion to compensate for this type of increase in insulin resistance [4, 5]. Asians also exhibit higher levels of endogenous basal glucose production, which suggests that this population has higher levels of hepatic insulin resistance [6]. Moreover, because Asians are less likely to develop hyperinsulinemia, they are more susceptible to the development of type 2 diabetes. These differences may be related to *β*-cell mass because Asian patients with type 2 diabetes, especially Koreans and Chinese, have a lower *β*-cell mass [5]. Aging is also an important contributor to this process. Thus, the identification and development of herbs that can attenuate insulin resistance as well as potentiate *β*-cell function and mass are becoming increasingly important, because these herbs can be ingested as food and/or drinks that may prevent the development of type 2 diabetes.

Mistletoe (*Viscum album coloratum*) is a general name for the woody parasites of several plant families, and most genera of Korean mistletoe belong to the family Santalaceae. Korean mistletoe tends to grow on oak trees and has traditionally been used as a herbal medicine in European, African, and Asian countries, including Korea. Korean mistletoe extract in water (KME-W) and Korean mistletoe extract in ethanol (KME-E) are not associated with any toxicity [7], and it has been confirmed that mistletoe is characterized by immunomodulatory, antidiabetic, antimicrobial, anticarcinogenic, antioxidant, and hypolipidemic activities [8–10]. This plant also contains various terpenoids, alkaloids, lectins, viscotoxins, phenylpropanoids, tannins, lignans, and polyphenols [11, 12]; however, its content varies somewhat based on the host trees from which they are collected and the area in which they grow. Similarly, the components and bioactivities of various mistletoe extracts differ according to the extraction solvents used to produce the solution. For example, triterpene acids have been quantified in aqueous mistletoe extracts (pH: 7.3), and the analyses revealed that oleanolic acid (1.1 *μ*g/mL) and betulinic acid (0.9 *μ*g/mL) are extracted with yields of less than 5% [12]. Furthermore, a high-performance liquid chromatography (HPLC) analysis conducted by our research group [10] demonstrated that KME-E contains betulin, betulinic acid, and oleanolic acid but that KME-W does not. On the other hand, KMW-W contains lectins and viscothionins [10]. These findings indicate that two different extracts of mistletoe possess different bioactive components. This result suggests that these compounds should be assessed to identify differences in their antidiabetic activities.

Thus, the present study aimed to determine whether the long-term consumption of KME-W or KME-E would have different antidiabetic activities in an animal model of nonobese type 2 diabetic rats and whether any differences would be associated with the primary terpenoids (betulin, betulinic acid, and oleanolic acid) found in the mistletoe extracts. These hypotheses were tested using partially pancreatectomized (Px) rats that received a high-fat diet. Additionally, the mechanisms underlying the antidiabetic activities of the extracts were assessed via the investigation of betulin, betulinic acid, and oleanolic acid in adipocytes and insulinoma cells. The primary aim of the present study was to determine which elements among the major terpenoids (betulin, betulinic acid, and oleanolic acid) in the extracts were most prominent and aided in the attenuation of insulin resistance and the potentiation of insulin secretion and cell proliferation.

## 2. Materials and Methods

### 2.1. Water Extracts of Mistletoe and Terpenoid Contents

Fresh mistletoe grown on oak trees was collected in January 2011 from Taebaek mountain in Gangwon-Do, Republic of Korea, and stored at −70°C until used. To prepare the mistletoe extract, mistletoe (100 g) was washed, dried at room temperature, freeze-dried, and powdered. The powder was extracted in distilled water at 100°C for 12 h or in 70% ethanol at 70°C for 12 h and each of them was centrifuged at 10,000 g at 4°C for 20 min. The supernatants were lyophilized in freeze-dryer.

The contents of total phenolic compounds in water or 70% ethanol extracts were measured using Folin-Ciocalteu reagent and expressed as mg gallic acid equivalents · g^−1^. The contents of total flavonoids were measured by the modified methods reported by modified Davis method and rutin was used as the standard. Each of free-dried extracts was dissolved in 70% ethanol and it had syringe filter to remove the undissolved contents. Terpenoids in each extract were analyzed by HPLC using Luna C18 column (4.6 mm × 250 mm ID 5 *μ*m). The mobile phase solvents consisted of acetonitrile and 0.2% acetic acid in water (8 : 2, v : v) with isocratic elution with a flow rate of 0.5 mL/min and 40°C in column temperature and UV detection was at 210 nm. The terpenoid contents were calculated from each of the standards such as betulinic acid, oleanolic acid, and betulin.

### 2.2. Animals and Ethics

Eight-week-old male Sprague-Dawley rats (weighing 218 ± 23 g) were housed individually in stainless steel cages in a controlled environment (23°C and with a 12 h light/dark cycle). All surgical and experimental procedures were performed according to the guidelines of the Animal Care and Use Review Committee of Hoseo University, Korea (2013-01). The rats had a 90% pancreatectomy using the Hosokawa technique [13] or received a sham pancreatectomy (Sham) under anesthesia induced by intramuscular injection of a mixture of ketamine and xylazine (100 and 10 mg/kg body weight, resp.). Px rats exhibited characteristics of type 2 diabetes (random glucose levels over 180 mg/dL), whereas the Sham rats did not [13, 14].

### 2.3. Experimental Design

The dosage of KME in the present study was based on a previous study [10] that provided diets containing 0.2% or 0.6% of 70% KME-E or KME-W to Px rats. Our preliminary study has demonstrated that the low dosage (0.5–2 *μ*g/mL) treatment with Korean mistletoe water extracts lower tumor necrosis factor-*α* expression in a dose-dependent manner in RAW 264.7 cells activated with lipopolysaccharides but higher dosage up to 10 *μ*g/mL does not change the efficacy [10]. In the present study, 30 Px rats were randomly assigned to the following three groups that differed according to diet: (1) 0.6% of KMW-W, (2) 0.6% of 70% KME-E, and (3) 0.6% of dextrin (Px-control). Additionally, 10 sham-operated rats (normal-control) received a high-fat diet containing 0.6% dextrose. All experimental animals were given free access to water and a high-fat diet containing either the assigned extracts or dextrose over the 8-week experimental period. The high-fat diet was a modified semipurified AIN-93 formulation for experimental animals [15] that consisted of 40% energy from carbohydrates, 20% energy from protein, and 45% energy from fats. The major carbohydrate, protein, and fat sources were starch and sugar, casein (milk protein), and lard (CJ Co., Seoul, Korea), respectively.

Overnight fasted serum glucose levels, food and water intakes, and body weight were measured every Tuesday at 10 AM. An oral glucose tolerance test (OGTT) was performed every three weeks in overnight fasted animals by orally administering 2 g glucose/kg body weight. Serum glucose and insulin were measured by tail bleeding at 0, 10, 20, 30, 45, 60, 90, and 120 min after glucose loading. Serum glucose levels were analyzed with a Glucose Analyzer II (Beckman, Palo Alto, CA), and serum insulin and leptin levels were measured by radioimmunoassay kit (Linco Research, Billerica, MA). Serum alanine aminotransferase (ALT) and aspartate aminotransferase activity (AST) were measured by standard colorimetric methods using commercial kits (Asan Pharmaceutical, Seoul, Republic of Korea).

### 2.4. Hyperglycemic Clamp

After seven weeks of the treatment, catheters were surgically implanted into the right carotid artery and left jugular vein of conscious and overnight fasted ten rats from each group after the anesthetization with ketamine and xylazine. After 5-6 days of implantation, a hyperglycemic clamp was performed in free-moving and overnight fasted rats to determine insulin secretion capacity as described in previous studies [13, 16, 17]. During the clamp, glucose was infused to maintain serum glucose levels of 5.5 mM above baseline and serum insulin levels were measured at designated time. After the clamp, rats were freely provided with foods and water for 2 days and next day they were deprived of food for 16 hours. The rats were anesthetized with the mixture of ketamine and xylazine and human regular insulin (5 U/kg body weight) was injected through the inferior vena cava of the rats. Ten min later, they were killed by decapitation and tissues were rapidly collected, frozen in liquid nitrogen, and stored at −70°C for further experiments. In order to determine the glycogen content in the liver, its lysates were centrifuged at 3000 rpm for 10 minutes and the supernatants deproteinized with 1.5 N perchloric acid. The glycogen content was calculated from glucose concentrations derived from glycogen hydrolyzed by *α*-amyloglucosidase in an acid buffer [18]. Triglyceride was extracted with a chloroform-methanol (2 : 1, vol/vol) from the liver and resuspended in pure chloroform [19]. Triacylglycerol concentration was determined using a Trinder kit (Young Dong Pharm., Seoul, Korea).

### 2.5. Euglycemic Hyperinsulinemic Clamp

After the catheterization of the right carotid artery and left jugular vein at the seventh week of the experimental periods, a euglycemic hyperinsulinemic clamp was performed on fasted conscious rats to determine insulin resistance as previously described [19, 20]. [3-^3^H] glucose (NEN Life Science, Boston, MA) was continuously infused during a 4-hour period at the rate of 0.05 *μ*Ci/min. Basal hepatic glucose output was measured in blood collected at 100 and 120 minutes after initiation of the [3-^3^H] glucose infusion. Then a primed continuous infusion of human regular insulin (Humulin, Eli Lilly, Indianapolis, IN) was initiated at a rate of 20 pmol × kg^−1^  × min^−1^ to raise plasma insulin concentration to approximately 1100 pM at 210–240 min. Blood samples from arteries were collected at 10-minute intervals for glucose estimation, and 25% glucose was infused at variable rates as needed to clamp glucose levels at approximately 6 mM. For the determination of plasma [3-^3^H] glucose concentrations, plasma was deproteinized with ZnSO_4_ and Ba(OH)_2_, dried to remove ^3^H_2_O, and resuspended in water, and disintegrations per min (dpm) of ^3^H were recorded. The plasma concentration of ^3^H_2_O was determined by the difference between ^3^H counts without and with drying. Rates of whole body glucose uptake and basal glucose turnover were determined as the ratio of the [^3^H] glucose infusion rate to the specific activity of plasma glucose (dpm/*μ*mol) during the final 30 minutes of the respective experiments. Hepatic glucose production at hyperinsulinemic clamped state was determined by subtracting the glucose infusion rate from the whole body glucose uptake.

### 2.6. Immunoblot Analysis

The liver collected from rats stimulated with insulin for 10 min was lysed with lysis buffer containing a 20 mM Tris buffer (pH 7.4) containing 2 mM EGTA, 137 mM NaCl, 1% NP40, 10% glycerol, and 12 mM *α*-glycerol phosphate and protease inhibitors. After 30 min on ice, the lysates were centrifuged for 10 min at 12,000 rpm at 4°C. After measuring protein contents in lysate by Bio Rad protein assay kit (Hercules, CA), lysates with equivalent amounts of protein (30–50 *μ*g) were resolved with SDS-PAGE and immunoblotted with antibodies of phospho-Akt^ser478^, Akt, phospho-glycogen synthase kinase- (GSK-) 1*β*, GSK-1*β* (Cell Signaling Technology, Beverly, MA), and phosphoenolpyruvate carboxykinase (PEPCK), generously provided by Dr. Garner of Vanderbilt University [16, 19]. The intensity of protein expression was determined using ImageQuant TL (Amersham Biosciences, Piscataway, NJ). These experiments were repeated three times for each group.

### 2.7. In Vitro Insulin-Stimulated Glucose Uptake and Insulin Signaling

Insulin-stimulated glucose uptake was analyzed by measuring the uptake of 2-deoxy-D-[^3^H] glucose in 3T3-L1 adipocytes, as previously described [21]. Briefly, the adipocytes were seeded in 24-well plates (4 × 10^4^ cells per well) in high glucose Dulbecco's Modified Eagle's Medium (DMEM; Invitrogen, Carlsbad, CA) containing fetal bovine serum (FBS) for 6 hours. The media were switched to low glucose DMEM (Invitrogen) containing 0.3% bovine serum albumin (BSA) and two concentrations (5 and 50 *μ*M) of betulin, betulinic acid, or oleanolic acid; they were then incubated for 16 hours at 37°C. The antidiabetic drug rosiglitazone (0.5 or 2 *μ*M) was used as a positive control. The media were then switched to a Krebs-Ringer-Hepes buffer (KRH) containing the respective compounds and either 0.2 or 10 nM insulin and incubated for 30 minutes at 37°C. Following the incubation period, glucose uptake was measured for 10 minutes using 0.1 *μ*Ci 2-deoxy-D-[^3^H] glucose and 1 mM glucose as the final concentrations. Nonspecific glucose uptake was measured following treatment with the extracts in the absence of insulin, and the radioactivity retained by the cell lysates was determined with a Wallac Liquid Scintillation Counter (Perkin Elmer, Waltham, MA).

After treatment with 50 *μ*M of betulin, betulinic acid, or oleanolic acid for 16 hour, the cells were lysated with the lysis buffer and then immunoblotted with antibodies for phospho-Akt^ser478^, Akt, phospho-GSK-1*β*, GSK-1*β*, phospho-AMPK, and AMPK (Cell Signaling Technology).

### 2.8. Peroxisome Proliferator-Activated Receptor- (PPAR-) *γ* Agonist Activity

Human embryonic kidney 293 cells were transiently transfected with a peroxisome proliferator responsive element- (PPRE-) luciferase construct (firefly pGL3-DR-1-luciferase; 0.12 *μ*g DNA·well^−1^), pSV-SPORT-PPAR-*γ* expression vector (0.12 *μ*g DNA·well^−1^), pSV-SPORT-retinoid X receptor-*α* vector (0.08 *μ*g DNA·well^−1^), and renilla phRL-TK vector (10 ng DNA·well^−1^) with a Lipofectamine PLUS reagent (Invitrogen, Carlsbad, CA) according to the manufacturer's protocol. PPAR-*γ* activity was measured as described in previous studies [21, 22]. After 2 h of transfection, vehicles (DMSO) or 5 or 50 *μ*M betulinic acid, oleanolic acid, or betulin was added to media for 40 h and the media were changed to serum-free DMEM containing 0.1% BSA, which also contained the respective extracts, for 12 h [22]. Both firefly (PPRE-luciferase) and renilla luciferase activities were measured using the Dual-Luciferase Reporter Assay System (Promega) in an Aureon PhL luminometer (Aureon Biosystems, Vienna, Austria). Ratios of firefly luciferase activity and renilla luciferase activity were calculated.

### 2.9. Glucose-Stimulated Insulin Secretion and Cell Viability

Mouse insulinoma cells (Min6 cells) were grown as previously described by Park et al. [22] in a 24-well plate at 6 × 10^4^ cells per well with high glucose DMEM containing 0.3% BSA and either vehicle or 5 or 50 *μ*m of betulinic acid, oleanolic acid, or betulin for 16 h. Exendin-4 (2.5 nM, Sigma Co., St. Louis, MO) treated cells were used as a positive control. After washing the cells with PBS, the Min6 cells were treated with vehicle or respective terpenoids in low (2 mM) or high glucose (20 mM) KRH buffer containing 20 mM Hepes pH 7.4 for 30 min. Insulin concentrations in supernatants from all treatments were measured using a radioimmunoassay kit (Linco Research, St. Charles, MO) and a Packard Cobra gamma-counter (Packard Instrument Co. Inc., Meriden, CT).

Cell viability was measured in Min6 cells treated with vehicle (DMSO) or 5 or 50 *μ*m of betulinic acid, oleanolic acid, and betulin and 2.5 nM of exendin-4 with a Cell Proliferation WST-1 reagent assay kit from Roche Diagnostic Co. (Indianapolis, IN) in an Aureon plate reader (Aureon Biosystems, Vienna, Austria). The kit is a modified tetrazolium salt that can be cleaved by metabolically active cells to a water soluble formazan, which was quantitated at 450 nm with an ELISA plate reader [23].

### 2.10. Statistical Analyses

All data are expressed as means ± standard deviations (SDs), and all statistical analyses were performed using SAS version 9.1 (SAS Institute, Cary, NC). In the animal studies, significant differences among the Px-control, KME-W, KME-E, and normal-control groups were identified with a one-way analysis of variance (ANOVA). In the cell-based studies, significant differences among the control, single compound, and positive control conditions were also identified with a one-way ANOVA. In both sets of studies, significant differences in the main effects among the groups were identified using post hoc Tukey's tests. A *P* value < 0.05 was considered to indicate statistical significance.

## 3. Results

### 3.1. Total Phenolic Compounds and Flavonoids

The 70% KME-E contained 1.7- and 2.7-fold higher levels of total polyphenols and flavonoids, respectively, than the KME-W. Additionally, the KME-E contained betulin, betulinic acid, and oleanolic acid, whereas KME-W did not (Table 1).

### 3.2. Energy Metabolism

During the 8-week experimental period, the Px-control group gained less body weight and had a lower epididymal fat mass than the normal-control group (Table 2). However, the caloric intake was higher in the Px-control than in the normal-control group even though their daily energy expenditures did not differ. This suggests that the lesser amount of weight gained by the Px-control rats was related to urinary glucose loss. The KME-W and KME-E groups had a tendency to gain more weight than the Px-control group, but these differences were not significant (Table 2). The daily energy intakes and energy expenditures of the KME-W and KME-E groups did not significantly differ from each other, but the cumulative energy intake of the KME-W group was lower than that of the KME-E group. Thus, treatment with KME-W and KME-E may reduce the urinary loss of glucose.

Interestingly, the epididymal fat masses of the KME-W and KME-E groups were lower than that of the Px-control group (Table 2); in fact, the fat mass serum leptin levels were highest in the normal-control group and descended in the order of the Px-control, KME-W, and KME-E groups. These differences were likely related to the energy sources used by the body. Rats in the Px-control and normal-control groups typically used fat and carbohydrates as their primary energy sources, whereas rats in the KME-W and KME-E groups used fat as their main energy source (Table 2). Thus, diabetic Px rats exhibited a somewhat deteriorated energy metabolism compared with the nondiabetic normal-control (sham) rats. Both KME-W and KME-E prevented this impairment in diabetic rats, albeit in an incomplete manner.

Since KME may have potential adverse effects on liver, serum ALT and AST activities which are known indicators of liver damage were measured in the present study. The levels were in a normal range and they rather decreased in KME-W and KME-E treatment. They were not significantly different in diabetic and nondiabetic controls.

### 3.3. Glucose Tolerance

**Figure 1:**
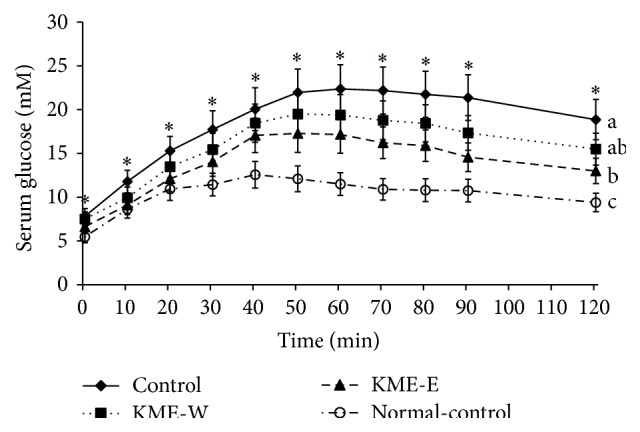
Changes in serum glucose concentrations during the oral glucose tolerance test. Oral glucose tolerance tests were performed on Px rats fed high-fat diets supplemented with either (1) 0.6% water extract of Korean mistletoe (KMW-W), (2) 0.6% of 70% ethanol extract of Korean mistletoe (KME-E), or (3) 0.6% dextrose (Px-control). As a normal-control group, sham-operated rats were provided with a high-fat diet containing 0.6% dextrose. After 8 weeks of treatment, glucose (2 g/kg body weight) was administered orally, blood samples were taken at the indicated time points, and serum glucose levels were measured. The sample size of each group was the same as in Table 2. The dots and error bars represent mean ± SD.  ^a,b,c^Values on the bars with different superscripts were significantly different in a Tukey post hoc test at a significance of *P* < 0.05.

Following an overnight fast, the serum glucose level of the Px-control group was significantly higher than that of the nondiabetic normal-control group, whereas the normal-control group had a significantly higher serum insulin level than the Px-control group. These results were related to the partial removal of the pancreas. After glucose loading, the serum glucose levels of the diabetic groups increased for 50 minutes, but they peaked at 40 minutes in the nondiabetic groups (Figure 1); these levels were lowest in the Px-control group and decreased in the order of the KME-W, KME-E, and normal-control groups. However, at the peak, the serum glucose level of the KME-E group was not as low as that of the normal-control group (Figure 1). The results of oral glucose tolerance test (OGTT) suggest that glucose-stimulated insulin secretion and insulin sensitivity may have been modified by the ingestion of KME-W and KME-E.

### 3.4. Glucose-Stimulated Insulin Secretion by Hyperglycemic Clamp

**Figure 2:**
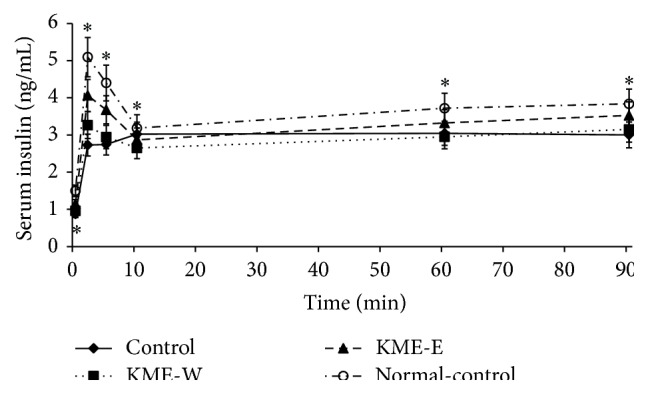
Insulin secretion capacity during hyperglycemic clamp. At the end of the experimental periods, hyperglycemic clamp was performed on Px rats fed high-fat diets supplemented with either (1) 0.6% water extract of Korean mistletoe (KMW-W), (2) 0.6% of 70% ethanol extract of Korean mistletoe (KME-E), or (3) 0.6% dextrose (Px-control). As a normal-control group, sham-operated rats were provided with a high-fat diet containing 0.6% dextrose. During hyperglycemic clamp, serum insulin levels were measured in free-moving and overnight-fasted diabetic rats as serum glucose levels at 5.5 mM above fasting levels were maintained. The sample size in each group was the same as in Table 3. The dots and error bars represent mean ± SD. ^*∗*^Significantly different among the groups in one-way ANOVA at a significance of *P* < 0.05.

In the present study, the glucose-stimulated insulin secretion capacity indicated the presence of *β*-cell function. During the hyperglycemic clamp procedure, serum insulin levels peaked between 2 and 5 minutes after the infusion of glucose into the jugular vein and then declined to a nadir at 10 minutes; this is referred to as first-phase insulin secretion. The serum glucose levels were sustained at more than 100 mg/dL above the baseline serum glucose levels for 60–90 minutes, and the serum insulin levels also increased from their nadir at 10 minutes and were then maintained at certain levels, known as second-phase insulin secretion (Figure 2). The diabetic Px-control group exhibited insulin levels that were approximately 59% and 81% of the first- and second-phase insulin secretion levels, respectively, of the nondiabetic normal-control group (Table 2). These findings indicate that the reduction of *β*-cell mass following the removal of the pancreas primarily resulted in a reduction in first-phase insulin secretion. KME-E, but not KME-W, prevented these reductions during the first- and second-phase insulin secretion in the Px-control group, but it did not do so to the same degree as it did in the normal-control group (Table 2).

The glucose infusion rates that were necessary to maintain serum glucose levels at 5.5 mM above baseline levels during the hyperglycemic clamp were lower in the Px-control group than in the normal-control group, whereas insulin sensitivity during the hyperglycemic state decreased in the Px-control group (Table 3). During the hyperglycemic state, KME-E increased the glucose infusion rates and insulin sensitivity to levels higher than those found in the Px-control group but lower than those observed in the normal-control group, whereas KME-W enhanced insulin sensitivity as much as KME-E (Table 3). These results suggest that KME-E promotes glucose-stimulated insulin secretion capacity and insulin sensitivity under hyperglycemic conditions in diabetic rats and that KME-W enhanced insulin sensitivity but does not influence insulin secretion capacity.

### 3.5. *β*-Cell Mass

Insulin secretion capacity is associated with pancreatic *β*-cell mass [24, 25]. In the present study, pancreatic *β*-cell mass was calculated by multiplying the weight of the pancreas by the *β*-cell area. We found that the Px-control group had a greater *β*-cell area than the normal-control group, and this increase was associated with a remarkable expansion of *β*-cell mass due to the role of insulin as the pancreas was regenerated. Due to removal of the pancreas, the pancreatic *β*-cell mass of the Px-control group was 56.4% of that of the normal-control group. The KME-E group, but not the KME-W group, exhibited an increase in *β*-cell area and *β*-cell mass compared with the Px-control group, but this increase was not as substantial as it was in the normal-control group (Table 4). This increase in *β*-cell area was associated with the number and size of individual *β*-cells. Indeed, individual *β*-cells were larger in size in the Px-control group than in the normal-control group, which indicates that there were fewer cells in the Px-control than in the normal-control group in the same-sized *β*-cell area (Table 4). This was confirmed by assessing *β*-cell proliferation and apoptosis. *β*-cell proliferation was greater in the normal-control group than in the Px-control group, but apoptosis was much higher in the Px-control group, which means that the Px-control group had fewer *β*-cells.

Both the KME-W and KME-E groups showed smaller-sized individual *β*-cells relative to those in the Px-control group. On the other hand, the KME-E, but not the KME-W, group showed increased proliferation of *β*-cells compared with the Px-control group, whereas both the KME-W and KME-E groups exhibited reduced *β*-cell apoptosis (Table 4). These results suggest that KME-E increased *β*-cell mass by hyperplasia and could maintain insulin secretion and sufficiently compensate for insulin resistance by sustaining *β*-cell function.

### 3.6. Insulin Sensitivity during the Euglycemic Hyperinsulinemic Clamp

**Figure 3:**
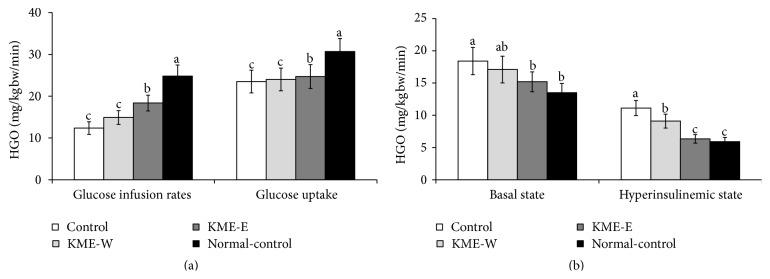
Serum glucose and insulin levels and glucose infusion rates during a euglycemic hyperinsulinemic clamp. Px rats fed high-fat diets supplemented with either (1) 0.6% water extract of Korean mistletoe (KMW-W), (2) 0.6% of 70% ethanol extract of Korean mistletoe (KME-E), or (3) 0.6% dextrose (Px-control). As a normal-control group, sham-operated rats were provided with a high-fat diet containing 0.6% dextrose. After an 8-week treatment period, the animals had a euglycemic hyperinsulinemic clamp assay in conscious, free-moving, and overnight fasted rats to determine whole body and hepatic insulin resistance. Glucose infusion rates and glucose uptake at a clamped steady-state (a) and hepatic glucose output at baseline and hyperinsulinemic state about 1100 pM of serum insulin (b) were measured. The sample size in each group was the same as in Table 3. The bars and error bars represent mean ± SD.  ^a,b,c^Values on the bars with different superscripts were significantly different in a Tukey post hoc test at a significance of *P* < 0.05.

During the euglycemic hyperinsulinemic clamp, measures of whole-body glucose utilization indicated the presence of whole-body insulin resistance under euglycemia. During the clamp, insulin was infused into the jugular vein to achieve serum insulin levels of approximately 1100 pM, and the infused glucose resulted in insulin levels of 100 mg/dL or a euglycemic state. At approximately 1100 pM, the glucose infusion rate and whole-body glucose uptake were lower in the diabetic Px-control group than in the nondiabetic normal-control group (Figure 3(a)). Glucose uptake was altered by both KME-E and KME-W, but glucose infusion rates under the hyperinsulinemic state increased to the greatest degree in the control group; this was followed by the KME-W, KME-E, and normal-control groups, in ascending order (Figure 3(a)). Hepatic glucose output levels under the basal and hyperinsulinemic states were not suppressed in the diabetic Px-control group relative to the nondiabetic normal-control group (Figure 3(b)). KME-E, but not KME-W, reduced hepatic glucose output under the basal state by approximately 27.4% relative to the comparable figure in the Px-control group. In a hyperinsulinemic state, the hepatic glucose output of the Px-control group showed a 1.9-fold increase compared with that of the normal-control group, whereas KME-E and KME-W suppressed this increase by 18.2% and 43.0%, respectively. The KME-E-induced suppression resulted in values similar to those of the normal-control group (Figure 3(b)).

### 3.7. Hepatic Insulin Signaling

**Figure 4:**
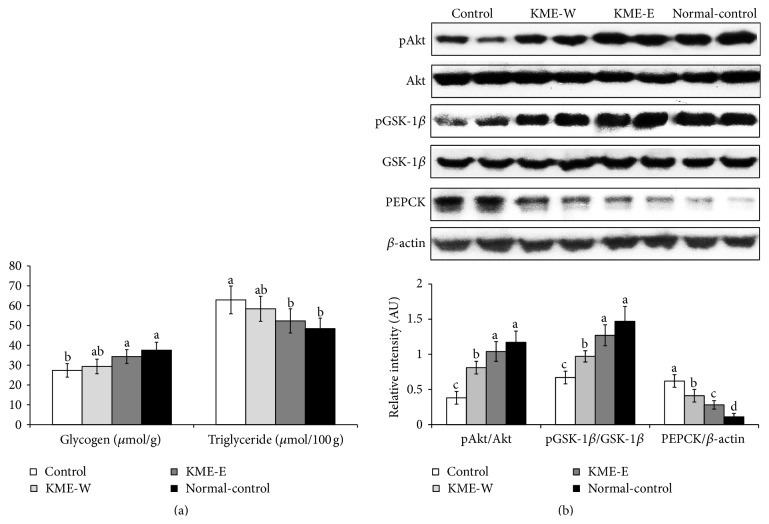
The modulation of insulin signaling in the liver at the end of experimental periods. After 10 min of insulin (5 U/kg body weight) stimulation through the inferior vena cava at the end of each experimental period, the liver was collected from the Px rats fed high-fat diets supplemented with either (1) 0.6% water extract of Korean mistletoe (KMW-W), (2) 0.6% of 70% ethanol extract of Korean mistletoe (KME-E), or (3) 0.6% dextrose (Px-control) and sham-operated rats fed a high-fat diet containing 0.6% dextrose (a normal-control). The liver was immediately lysed with a lysis buffer. Hepatic glycogen and triglyceride contents were also determined (a). The phosphorylation and expression levels of the Akt, GSK-1*β*, and PEPCK, involved in insulin sensitivity, were determined by immunoblotting with specific antibodies. The intensity of protein expression was determined using ImageQuant TL (b). These experiments were repeated four times for the liver, and the bars and error bars represent mean ± SD (*n* = 4).  ^a,b,c,d^Values on the bars with different superscripts were significantly different in a Tukey post hoc test at a significance of *P* < 0.05.

The diabetic Px-control group had less glycogen storage but increased triglyceride content in the liver compared with the nondiabetic normal-control group. KME-E prevented the deterioration of hepatic glycogen and triglyceride storage (Figure 4(a)), and these changes were related to hepatic insulin signaling, which was determined by assessing the phosphorylation of Akt and GSK-1*β* and the expression of PEPCK in the liver. The serine phosphorylation rates of Akt and GSK-1*β* were attenuated in the Px-control group compared with the normal-control group (Figure 4(b)). This phosphorylation was potentiated in the ascending order of KME-W and KME-E and, moreover, the phosphorylation was increased in the KME-E group as much as in the normal-control group. In contrast to Akt phosphorylation, PEPCK expression was higher in the Px-control group than in the normal-control group, and it was reduced in the KME-E group (Figure 4(b)).

### 3.8. Insulin-Stimulated Glucose Uptake in 3T3-L1 Adipocytes and PPAR-*γ* Activity

**Figure 5:**
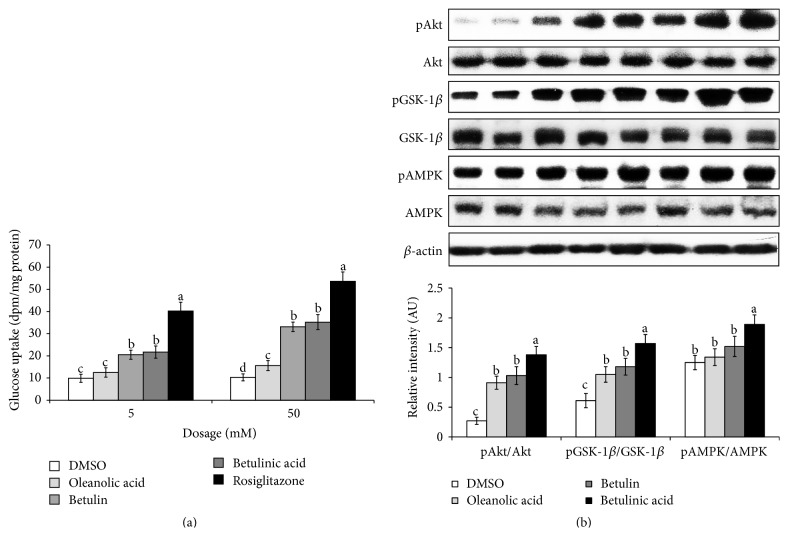
Insulin-stimulated glucose uptake in 3T3-L1 adipocytes and PPAR-*γ* activity by betulin, betulinic acid, and oleanolic acid. 3T3-L1 adipocytes were treated with low (0.5 *μ*g/mL) or high (5 *μ*g/mL) doses of vehicle (DMSO) or low (5 *μ*M) or high (50 *μ*M) doses of betulin, betulinic acid, and oleanolic acid for 16 hours. At the end of the incubation, insulin (0.2 nM) was administered for 30 minutes to determine insulin-stimulated glucose uptake, which would represent insulin-sensitizing activities. Vehicle and 10 nM insulin treatments were used as negative and positive controls, respectively. The data are presented as ^3^H-deoxyglucose content per mg of protein in the cells, which indicates the degree of glucose uptake (*n* = 7). After 16 hours of treatment with betulin, betulinic acid, or oleanolic acid, the cells were immediately lysed with a lysis buffer, and the phosphorylation and expression levels of Akt, GSK-1*β*, and AMPK were determined by immunoblotting with specific antibodies (b).  ^a,b,c,d^Values on the bars with different superscripts were significantly different in a Tukey post hoc test at a significance of *P* < 0.05.

The most likely effective components in the KME solutions were betulin, betulinic acid, and oleanolic acid, and the insulin-sensitizing effects of these compounds were tested via treatment with a low dose of insulin (0.2 nM). Following administration of 0.2 nM of insulin, glucose uptake was 10.1 ± 1.4 disintegrations per minute (dpm)/*μ*g protein. Furthermore, betulin concentrations of 5 and 50 mM increased insulin-stimulated glucose uptake by 2.1- and 3.2-fold, respectively, but these increases were less than those induced by rosiglitazone treatment. Treatment with betulinic acid also elevated insulin-stimulated glucose uptake in a dose-dependent manner but to a lesser degree than betulin treatment. In contrast, oleanolic acid only minimally enhanced insulin-stimulated glucose uptake. Thus, betulin and betulinic acid exhibited moderate insulin-sensitizing activity (Figure 5(a)). All of betulin, betulinic acid, and oleanolic acid improved insulin-stimulated insulin signaling (the phosphorylation of Akt and GSK-1*β*) in 3T3-L1 adipocytes and betulinic acid potentiated it the most (Figure 5(b)). In addition, betulinic acid enhanced the phosphorylation of AMPK (Figure 5(b)).

**Figure 6:**
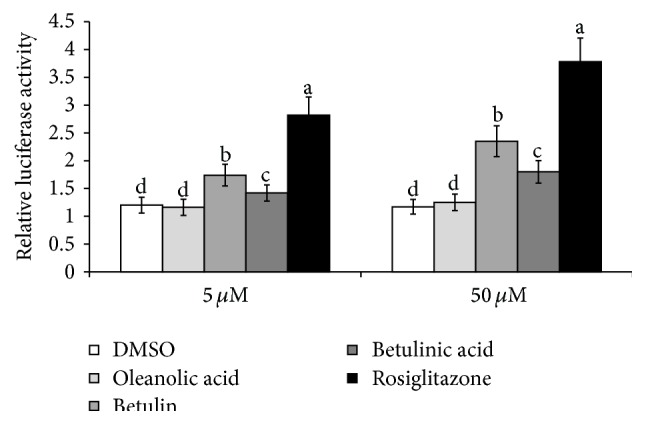
PPAR-*γ* activity of betulin, betulinic acid, and oleanolic acid as determined by luciferase ligands. The cells were transiently transfected with four vectors such as a PPRE-luciferase construct, pSV-SPORT-PPAR-*γ* expression vector, pSV-SPORT-retinoid X receptor- (RXR-) *α* vector, and renilla phRL-TK vector with a Lipofectamine PLUS reagent. After 2 h of transfection, the cells were treated with serum-free DMEM containing 0.1% BSA for 40 h and were exposed to betulin, betulinic acid, and oleanolic acid (5 and 50 *μ*M) for 12 hours. At the end of the incubation, the cells were solubilized in 1x passive lysis buffer and assayed for both firefly (PPRE-luciferase) and renilla luciferase activities using the Dual-Luciferase Reporter Assay System. Ratios of firefly luciferase activity and renilla luciferase activity were used for the results. The bars and error bars represent mean ± SD (*n* = 7).  ^a,b^Values on the bars with different superscripts were significantly different in a Tukey post hoc test at a significance of *P* < 0.05.

Betulin and betulinic acid increased PPAR-*γ* activity, but betulin was a more effective agonist of PPAR-*γ* activity than betulinic acid. However, the PPAR-*γ*-related agonistic activity of betulin was not as high as that of rosiglitazone (2 *μ*M), which is a known commercial PPAR-*γ* agonist (Figure 6). Thus, betulinic acid and betulin resulted in mild and moderate PPAR-*γ* agonistic activity, respectively.

### 3.9. Glucose-Stimulated Insulin Secretion and Cell Viability in Min6 Cells

**Figure 7:**
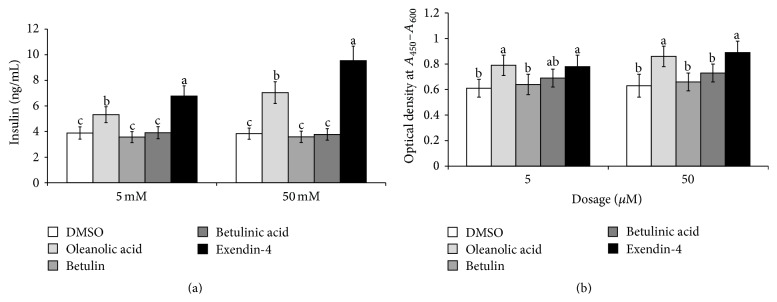
Glucose-stimulated insulin secretion and cell viability in insulinoma Min6 cells. Min6 insulinoma cells were treated with low (0.5 *μ*g/mL) or high (5 *μ*g/mL) doses of vehicle (DMSO) or low (5 *μ*M) or high (50 *μ*M) doses of betulin, betulinic acid, and oleanolic acid for 16 hours. At the end of the incubation, media were changed into high glucose (20 mM) Krebs-Ringer-Hepes buffer containing assigned compound for 30 min and insulin concentrations in the buffer were measured by RIA insulin kit (a). After treatment with vehicle (DMSO) or low (5 *μ*M) or high (50 *μ*M) doses of betulin, betulinic acid, and oleanolic acid for 48 h and glucose-stimulated insulin secretion measured for 30 min, cell viability was measured with a cell proliferation WST-1 reagent assay kit (b). The bars and error bars represent mean ± SD (*n* = 7).  ^a,b,c^Values on the bars with different superscripts were significantly different in a Tukey post hoc test at a significance of *P* < 0.05.

In insulinoma Min6 cells, insulin secretion was increased by 5.1 ± 0.7-fold in the high-glucose (20 mM) DMEM media compared with the low-glucose (2 mM) media. Betulin, betulinic acid, and oleanolic acid did not stimulate insulin secretion in the low-glucose media (data not shown), but oleanolic acid potentiated insulin secretion in dose-dependent manner in the high-glucose media. However, this increase was not to the same degree as that initiated by exendin-4 (2.5 nM), which is a known insulinotropic agent (Figure 7(a)).

Relative to the control group, oleanolic acid increased cell proliferation in a dose-dependent manner, and betulinic acid also enhanced cell proliferation, but it was less than that induced by 50 *μ*M of oleanolic acid (Figure 7(b)). Thus, oleanolic acid exerted insulinotropic action.

## 4. Discussion

KME-W has higher concentrations of lectins and viscothionins, which are associated with immune modulation, whereas KME-E has a concentration of triterpenoids, which are related to metabolic diseases. In the present study, triterpenoids were detected in KME-E but not KME-W, and this difference resulted in different antidiabetic activities in diabetic rats. Type 2 diabetes is induced when enhanced insulin secretion cannot compensate for increasing levels of insulin resistance. Thus, the tight regulation of insulin resistance and *β*-cell function play important roles in the prevention of type 2 diabetes as well as in delaying its progression.

The present findings demonstrated that, in a hyperglycemic clamp, glucose-stimulated insulin secretion, particularly first-phase insulin, was potentiated in the KME-E, but not in the KME-W, group compared with the Px-control group. KME-E also enhanced *β*-cell mass to a greater degree than observed in the Px-control group. Additionally, KME-E reduced whole-body insulin resistance relative to that observed in the Px-control group, whereas hepatic insulin resistance was attenuated in the Px-control, KME-W, and KME-E groups, in descending order. Therefore, KME-E prevented the deterioration of glucose metabolism in diabetic Px rats via the actions of betulin, betulinic acid, and oleanolic acid. Betulin potentiated insulin-stimulated glucose uptake by increasing PPAR-*γ* activities in 3T3-L1 adipocytes, whereas oleanolic acid enhanced glucose-stimulated insulin secretion and cell proliferation in Min6 insulinoma cells. Therefore, KME-E may be a potential therapeutic agent for patients with type 2 diabetes. To the best of our knowledge, no studies have evaluated the modulation of insulin secretion and insulin resistance in terms of antidiabetic activity.

KME has traditionally been used as a herbal medicine in European, African, and Asian countries, including Korea. Analyses of various crude alcoholic extracts and purified fractions of KME have revealed that these products possess hypotensive, hypoglycemic, antilipidemic, antioxidative, anti-inflammatory, and antimicrobial activities and tend to ameliorate health problems such as diabetes mellitus, hypertension, arthritis, pain, and cancer [26]. Adaramoye et al. [8] reported that 3 weeks of treatment with African mistletoe methanolic extract reduced fasting blood glucose concentrations and glycated hemoglobin (HbA1c) levels to the same degree as glibenclamide treatment in streptozotocin-induced diabetic rats. However, these findings did not reveal any changes in insulin secretion and insulin resistance. Orhan et al. [27] found that the antidiabetic activities of mistletoe extracts were highly dependent on the host plant species and the extraction solvents that are used. For example, these authors demonstrated that 7 days of treatment with an ethanolic extract of European mistletoe grown on pine trees produced the greatest reduction in serum glucose levels during an OGTT in normal and streptozotocin-induced diabetic rats. Furthermore, the maximum effects of a methanolic extract (400 mg/kg) of African mistletoe from* Persea americana*, the avocado tree, reduced blood glucose levels in alloxan-induced diabetic rats as much as glibenclamide treatment 24 hours after administration [28]. This study also found no toxicity among the mistletoe extracts obtained from five different host trees. The present study is the first to use KME grown on oak trees to investigate the antidiabetic activities of the extract. The majority of previous studies have used streptozotocin or alloxan to induce diabetes in experimental animals, and these compounds are known to destroy *β*-cells via the generation of free radicals [8, 27, 28]. Thus, mistletoe likely reduces blood glucose levels by reducing the level of free radicals and partly preventing *β*-cell damage.

In the present study, KME-E exerted superior antidiabetic activities relative to KME-W by potentiating *β*-cell function and mass and reducing insulin resistance in type 2 diabetic Px rats. These effects were most likely related to the actions of triterpenoids, such as betulin, betulinic acid, and oleanolic acid, which were present in KME-E. In the cell culture analyses of the present study, betulin and betulinic acid enhanced insulin-stimulated glucose uptake via PPAR-*γ* activity in adipocytes, whereas oleanolic acid potentiated glucose-stimulated insulin secretion in insulinoma cells. Consistent with these results, several studies have shown that betulin and betulinic acid improve glucose metabolism. Using a diet-induced obesity animal model, Tang et al. [29] demonstrated that betulin reduces the biosynthesis of cholesterol and fatty acids by inhibiting sterol regulatory element-binding protein (SREBP) pathways. It has also been shown that betulin dramatically enhances the expressions of adiponectin, lipoprotein lipase, and PPAR-*γ* in white adipose tissues, which may improve insulin sensitivity and glucose metabolism [29]. Additionally, Wan et al. [30] reported that betulin and betulinic acid suppress the expression of SREBP-1 and its target genes related to fatty acid synthesis in hepatocytes, which results in protection against acute ethanol-induced fatty liver. Similarly, betulinic acid alleviates nonalcoholic fatty liver disease by potentiating the AMPK/mammalian target of the rapamycin/SREBP signaling pathway in mice with diet-induced obesity [31]. In the present study, betulinic acid activated AMPK via phosphorylation in 3T3-L1 adipocytes. Thus, the betulin and betulinic acid components of KME-E may play important roles in the improvement of glucose metabolism by enhancing insulin sensitivity in the adipose tissues and liver of diabetic Px rats.

Type 2 diabetes is strongly related to pancreatic *β*-cell dysfunction, which is influenced by *β*-cell mass. In the present study, KME-E, but not KME-W, potentiated glucose-stimulated insulin secretion and *β*-cell proliferation in diabetic Px rats, and it was shown that this resulted from the oleanolic acid component of KME-E. Oleanolic acid exerts antidiabetic activities in diabetic animals; these activities are primarily explained by enhanced pancreatic *β*-cell function and reduced *β*-cell apoptosis [32, 33]. Additionally, oleanolic acid attenuates hepatic insulin resistance via antioxidant, hypolipidemic, and anti-inflammatory activities [34, 35]. As a result, oleanolic acid can ameliorate diabetic symptoms by potentiating *β*-cell function, resulting in improved insulin resistance, especially in the liver.

It has been suggested that KME is toxic to the liver due to several of its components, including lectins [7]. Preliminary findings from our research group have shown that KME is generally nontoxic when it is boiled for more than 10 hours because the toxic compounds may be denatured; we also found that KME-W contains lectins but that KME-E does not [10]. Therefore, KME-E has a much lower chance of inducing liver toxicity. Kim et al. [36] have also shown that extracts of mistletoe grown on oak tree have LD50 of above 5,000 mg/kg in rats and no extract-related adverse effects are revealed with up to 1,000 mg/kg body weight/day in rats of chronic consumption study. Korean mistletoe is accepted to be safe to consume. The present study also demonstrated that both KME-W and KME-E did not lead to an increase in aspartate aminotransferase and alanine aminotransferase activities in the blood, which indicates that the liver was not damaged by either of the extracts.

## 5. Conclusion

KME-E improved glucose tolerance via improvements in hepatic insulin resistance and alterations of *β*-cell function and mass in diabetic Px rats. The superior antidiabetic effects of KME-E compared with KME-W were likely due to the presence of betulin, betulinic acid, and oleanolic acid in the ethanolic extract. It was also shown that betulin and betulinic acid acted as PPAR-*γ* agonists and increased insulin-stimulated glucose uptake and potentiated the phosphorylation of AMPK in 3T3-L1 adipocytes. Furthermore, oleanolic acid enhanced *β*-cell function and mass. Therefore, KME-E can be a potential therapeutic agent for the treatment of patients with type 2 diabetes.

## Figures and Tables

**Table 1:** The contents of phenolic compounds and flavonoids (unit: mg/g dry weight).

	Water extract of Korean mistletoe	70% methanol extract of Korean mistletoe
Polyphenols	3.53 ± 0.54	5.86 ± 0.63
Flavonoids	1.32 ± 0.22	2.38 ± 0.31
Betulin	ND	0.11 ± 0.01
Betulinic acid	ND	0.51 ± 0.00
Oleanolic acid	ND	0.89 ± 0.01

Values are means ± SD. ND, nondetectable.

**Table 2:** Metabolic changes at the end of 8-week treatment.

	Control (*n* = 16)	KME-W (*n* = 16)	KME-E (*n* = 16)	Normal-control (*n* = 16)
Body weight (g)	337 ± 31^b^	342 ± 32^b^	358 ± 34^b^	407 ± 37^a^
Epididymal fat pads (g)	4.2 ± 0.6^b^	3.3 ± 0.6^c^	3.6 ± 0.5^c^	5.9 ± 0.8^a^
Relative epididymal fat pad (g/kg bw)	12.4 ± 1.7^b^	9.6 ± 1.4^c^	10.1 ± 1.6^c^	14.5 ± 1.9^a^
Caloric intakes (kcal/day)	128 ± 16^a^	118 ± 14^a^	117 ± 15^a^	101 ± 14^b^
Energy expenditure (kcal/kg^0.75^/day)	92.1 ± 12.1	97.9 ± 12.1	92.7 ± 11.6	94.3 ± 11.6
Carbohydrate oxidation (mg/kg^0.75^/min)	3.9 ± 0.5^a^	3.7 ± 0.5^ab^	3.4 ± 0.4^b^	4.2 ± 0.5^a^
Fat oxidation (mg/kg^0.75^/min)	5.9 ± 0.8^b^	6.8 ± 0.8^a^	6.5 ± 0.7^ab^	5.8 ± 0.7^b^
Overnight fasted serum leptin levels (ng/mL)	5.5 ± 0.8^b^	4.4 ± 0.7^c^	4.2 ± 0.8^c^	6.5 ± 0.9^a^
Overnight fasted serum glucose (mmol/L)	8.0 ± 1.0^a^	7.5 ± 0.9^a^	6.6 ± 0.8^b^	5.4 ± 0.6^c^
Overnight fasted serum insulin (ng/mL)	0.82 ± 0.14^b^	0.88 ± 0.15^b^	1.07 ± 0.19^a^	1.14 ± 0.20^a^
Serum AST (IU/L)	130 ± 13.0^a^	112 ± 12^b^	118 ± 13^b^	125 ± 13^a^
Serum ALT (IU/L)	45 ± 4.8^a^	36.4 ± 4.2^b^	38.4 ± 4.4^b^	44.2 ± 4.6^a^

Values are mean ± SD. Diabetic Px rats were fed with high-fat diets supplementing (1) 0.6% water extract of Korean mistletoe (KMW-W), (2) 0.6% of 70% ethanol extract (KME-E), or (3) 0.6% dextrose (Control). Sham-operated rats were provided with a high-fat diet containing 0.6% dextrose as a normal-control.

^a,b,c^Values in the same row with different superscripts were significantly different in Tukey test at *P* < 0.05.

**Table 3:** Insulin secretion capacity during hyperglycemic clamp.

	Control (*n* = 8)	KME-W (*n* = 8)	KME-E (*n* = 8)	Normal-control (*n* = 8)
Serum insulin at basal state (ng/mL)	0.84 ± 0.11^c^	0.90 ± 0.12^c^	1.09 ± 0.16^b^	1.46 ± 0.21^a^
Serum insulin at first phase (ng/mL)	2.74 ± 0.30	3.11 ± 0.35^c^	3.87 ± 0.43^b^	4.75 ± 0.51^a^
Serum insulin at second phase (ng/mL)	3.02 ± 0.31^b^	3.05 ± 0.32^b^	3.43 ± 0.36^a^	3.78 ± 0.40^a^
AUC of insulin at first phase (ng/mL*∗*min)	19.3 ± 2.1^c^	20.5 ± 2.3^c^	26.2 ± 3.1^b^	32.8 ± 3.6^a^
AUC of insulin at second phase (ng/mL*∗*min)	186 ± 20^bc^	176 ± 20^c^	202 ± 22^b^	230 ± 24^a^
Glucose infusion rate (mg/kg bw/min)	12.2 ± 1.5^c^	14.0 ± 1.7^bc^	16.2 ± 2.1^b^	26.5 ± 3.2^a^
Insulin sensitivity (*µ*mol glucose· min^−1^·100 g^−1^ per *µ*mol insulin/L)	10.9 ± 1.4^c^	13.3 ± 1.7^b^	13.4 ± 1.7^b^	19.2 ± 2.3^a^

Values are mean ± SD. Diabetic Px rats were fed with high-fat diets supplementing (1) 0.6% water extract of Korean mistletoe (KMW-W), (2) 0.6% of 70% ethanol extract (KME-E), or (3) 0.6% dextrose (Control). Sham-operated rats were provided with a high-fat diet containing 0.6% dextrose as a normal-control.

^a,b,c^Values in the same row with different superscripts were significantly different in Tukey test at *P* < 0.05.

**Table 4:** The modulation of islet morphometry at the end of experiment.

	Control (*n* = 6)	KME-W (*n* = 6)	KME-E (*n* = 6)	Normal-control (*n* = 6)
*β*-cell area (%)	7.5 ± 0.9^b^	7.7 ± 0.8^b^	8.7 ± 0.9^a^	6.3 ± 0.7^c^
Individual *β*-cell size (*μ*m^2^)	242 ± 30^a^	193 ± 25^b^	201 ± 24^b^	189 ± 23^b^
Absolute *β*-cell mass (mg)	32.3 ± 3.4^c^	33.5 ± 3.7^c^	39.8 ± 4.0^b^	57.3 ± 6.2^a^
BrdU^+^ cells (% BrdU^+^ cells of islets)	0.97 ± 0.13^a^	1.01 ± 0.13^b^	1.14 ± 0.15^a^	0.75 ± 0.11^c^
Apoptosis (% apoptotic bodies of islets)	1.08 ± 0.12^a^	0.92 ± 0.09^b^	0.88 ± 0.12^b^	0.73 ± 0.08^b^

Values are mean ± SD. Diabetic Px rats were provided with high-fat diets supplementing (1) 0.6% water extract of Korean mistletoe (KMW-W), (2) 0.6% of 70% ethanol extract (KME-E), or (3) 0.6% dextrose (Control). Sham-operated rats were provided with a high-fat diet containing 0.6% dextrose as a normal-control.

^a,b,c^Values in the same row with different superscripts were significantly different in Tukey test at *P* < 0.05.

## References

[1] Cheema A., Adeloye D., Sidhu S., Sridhar D., Chan K. Y. (2014). Urbanization and prevalence of type 2 diabetes in Southern Asia: a systematic analysis. *Journal of Global Health*.

[2] Nagao M., Asai A., Sugihara H., Oikawa S. (2015). Fat intake and the development of type 2 diabetes. *Endocrine Journal*.

[3] Park S., Park C. H., Jang J. S. (2006). Antecedent intake of traditional Asian-style diets exacerbates pancreatic *β*-cell function, growth and survival after Western-style diet feeding in weaning male rats. *Journal of Nutritional Biochemistry*.

[4] Kim C.-H., Kim H.-K., Kim E. H., Bae S. J., Park J.-Y. (2013). Relative contributions of insulin resistance and beta-cell dysfunction to the development of Type 2 diabetes in Koreans. *Diabetic Medicine*.

[5] Møller J. B., Man C. D., Overgaard R. V. (2014). Ethnic differences in insulin sensitivity, *β*-cell function, and hepatic extraction between Japanese and caucasians: a minimal model analysis. *Journal of Clinical Endocrinology and Metabolism*.

[6] Wium C., Gulseth H. L., Eriksen E. F., Birkeland K. I. (2013). Characteristics of glucose metabolism in Nordic and South Asian subjects with type 2 diabetes. *PLoS ONE*.

[7] Kienle G. S., Grugel R., Kiene H. (2011). Safety of higher dosages of *Viscum album* L. in animals and humans—systematic review of immune changes and safety parameters. *BMC Complementary and Alternative Medicine*.

[8] Adaramoye O., Amanlou M., Habibi-Rezaei M., Pasalar P., Ali M.-M. (2012). Methanolic extract of African mistletoe (*Viscum album*) improves carbohydrate metabolism and hyperlipidemia in streptozotocin-induced diabetic rats. *Asian Pacific Journal of Tropical Medicine*.

[9] Lee J. Y., Kim J. Y., Lee Y. G. (2007). In vitro immunoregulatory effects of Korean mistletoe lectin on functional activation of monocytic and macrophage-like cells. *Biological and Pharmaceutical Bulletin*.

[10] Kim M. J., Park J. H., Kwon D. Y. (2015). The supplementation of Korean mistletoe water extracts reduces hot flushes, dyslipidemia, hepatic steatosis, and muscle loss in ovariectomized rats. *Experimental Biology and Medicine*.

[11] López-Martínez S., Navarrete-Vázquez G., Estrada-Soto S., León-Rivera I., Rios M. Y. (2013). Chemical constituents of the hemiparasitic plant *Phoradendron brachystachyum* DC Nutt (Viscaceae). *Natural Product Research*.

[12] Jäger S., Winkler K., Pfüller U., Scheffler A. (2007). Solubility studies of oleanolic acid and betulinic acid in aqueous solutions and plant extracts of *Viscum album* L.. *Planta Medica*.

[13] Hosokawa Y. A., Hosokawa H., Chen C., Leahy J. L. (1996). Mechanism of impaired glucose-potentiated insulin secretion in diabetic 90% pancreatectomy rats. Study using glucagonlike peptide-1 (7–37). *The Journal of Clinical Investigation*.

[14] Islam M. S., Wilson R. D. (2012). Experimentally induced rodent models of type 2 diabetes. *Methods in Molecular Biology*.

[15] Reeves P. G., Nielsen F. H., Fahey G. C. (1993). AIN-93 purified diets for laboratory rodents: final report of the American Institute of Nutrition ad hoc writing committee on the reformulation of the AIN-76A rodent diet. *Journal of Nutrition*.

[16] Soo B. C., Jin S. J., Park S. (2005). Estrogen and exercise may enhance *β*-cell function and mass via insulin receptor substrate 2 induction in ovariectomized diabetic rats. *Endocrinology*.

[17] Dobbins R. L., Szczepaniak L. S., Myhill J. (2002). The composition of dietary fat directly influences glucose-stimulated insulin secretion in rats. *Diabetes*.

[18] Frontoni S., Choi S. B., Banduch D., Rossetti L. (1991). In vivo insulin resistance induced by amylin primarily through inhibition of insulin-stimulated glycogen synthesis in skeletal muscle. *Diabetes*.

[19] Kwon D. Y., Kim Y. S., Ryu S. Y. (2013). Capsiate improves glucose metabolism by improving insulin sensitivity better than capsaicin in diabetic rats. *Journal of Nutritional Biochemistry*.

[20] Kim J. K., Kim Y.-J., Fillmore J. J. (2001). Prevention of fat-induced insulin resistance by salicylate. *The Journal of Clinical Investigation*.

[21] Choi S. B., Wha J. D., Park S. (2004). The insulin sensitizing effect of homoisoflavone-enriched fraction in *Liriope platyphylla* Wang et Tang via PI3-kinase pathway. *Life Sciences*.

[22] Park S., Ahn I. S., Kim J. H., Lee M. R., Kim J. S., Kim H. J. (2010). Glyceollins, one of the phytoalexins derived from soybeans under fungal stress, enhance insulin sensitivity and exert lnsulinotropic actions. *Journal of Agricultural and Food Chemistry*.

[23] Yang H. J., Kwon D. Y., Moon N. R. (2013). Soybean fermentation with *Bacillus licheniformis* increases insulin sensitizing and insulinotropic activity. *Food & Function*.

[24] Ferrannini E., Mari A. (2014). Beta-cell function in type 2 diabetes. *Metabolism: Clinical and Experimental*.

[25] Ahren B. (2005). Type 2 diabetes, insulin secretion and *β*-cell mass. *Current Molecular Medicine*.

[26] Adesina S. K., Illoh H. C., Johnny I. I., Jacobs I. E. (2013). African mistletoes (*Loranthaceae*); ethnopharmacology, chemistry and medicinal values: an update. *African Journal of Traditional, Complementary, and Alternative Medicines*.

[27] Orhan D. D., Aslan M., Sendogdu N., Ergun F., Yesilada E. (2005). Evaluation of the hypoglycemic effect and antioxidant activity of three *Viscum album* subspecies (European mistletoe) in streptozotocin-diabetic rats. *Journal of Ethnopharmacology*.

[28] Osadebe P. O., Okide G. B., Akabogu I. C. (2004). Study on anti-diabetic activities of crude methanolic extracts of *Loranthus micranthus* (Linn.) sourced from five different host trees. *Journal of Ethnopharmacology*.

[29] Tang J.-J., Li J.-G., Qi W. (2011). Inhibition of SREBP by a small molecule, betulin, improves hyperlipidemia and insulin resistance and reduces atherosclerotic plaques. *Cell Metabolism*.

[30] Wan Y., Jiang S., Lian L.-H. (2013). Betulinic acid and betulin ameliorate acute ethanol-induced fatty liver via TLR4 and STAT3 in vivo and in vitro. *International Immunopharmacology*.

[31] Quan H. Y., Kim D. Y., Kim S. J., Jo H. K., Kim G. W., Chung S. H. (2013). Betulinic acid alleviates non-alcoholic fatty liver by inhibiting SREBP1 activity via the AMPK-mTOR-SREBP signaling pathway. *Biochemical Pharmacology*.

[32] Wang X., Chen H. L., Liu J. Z. (2013). Protective effect of oleanolic acid against beta cell dysfunction and mitochondrial apoptosis: crucial role of ERK-NRF2 signaling pathway. *Journal of Biological Regulators and Homeostatic Agents*.

[33] Teodoro T., Zhang L., Alexander T., Yue J., Vranic M., Volchuk A. (2008). Oleanolic acid enhances insulin secretion in pancreatic *β*-cells. *FEBS Letters*.

[34] Wang X., Liu R., Zhang W. (2013). Oleanolic acid improves hepatic insulin resistance via antioxidant, hypolipidemic and anti-inflammatory effects. *Molecular and Cellular Endocrinology*.

[35] Zeng X.-Y., Wang Y.-P., Cantley J. (2012). Oleanolic acid reduces hyperglycemia beyond treatment period with Akt/FoxO1-induced suppression of hepatic gluconeogenesis in type-2 diabetic mice. *PLoS ONE*.

[36] Kim I., Jeong J.-S., Yoon T. J., Kim J. B. (2013). Safety evaluation of Korean mistletoe extract. *The Korean Journal of Food and Nutrition*.

